# Response to sequential treatment with prednisolone and vigabatrin in infantile spasms

**DOI:** 10.1111/jpc.16181

**Published:** 2022-08-24

**Authors:** Winston Dzau, Sally Cheng, Penny Snell, Michael Fahey, Ingrid E Scheffer, A Simon Harvey, Katherine B Howell

**Affiliations:** ^1^ Department of Paediatrics University of Melbourne Melbourne Victoria Australia; ^2^ Department of Neurology The Royal Children's Hospital Melbourne Victoria Australia; ^3^ Neuroscience Research Group Murdoch Children's Research Institute Melbourne Victoria Australia; ^4^ Department of Paediatrics Monash University Melbourne Victoria Australia; ^5^ Department of Medicine University of Melbourne, Austin Health Melbourne Victoria Australia; ^6^ Florey Institute of Neurosciences and Mental Health Melbourne Victoria Australia

**Keywords:** infantile spasms, prednisolone, treatment, vigabatrin

## Abstract

**Aim:**

To report response to first treatment in infants with infantile spasms (IS), including incremental benefit of prednisolone 60 mg/day and vigabatrin following prednisolone 40 mg/day failure in infants commenced on the United Kingdom Infantile Spasms Study (UKISS) treatment sequence.

**Methods:**

In this retrospective analysis, we compared effectiveness of prednisolone, vigabatrin and nonstandard treatments as first treatment for IS. In infants who commenced the UKISS treatment sequence, we evaluated response to each step. Primary outcome was spasm cessation after 42 days. Secondary outcomes were severe side effects and spasm relapse after 42 days.

**Results:**

Treatment response data were available for 151 infants. First treatment was prednisolone in 99 infants, vigabatrin in 18 and nonstandard treatment in 34. The rate of spasm cessation with first treatment was significantly higher with prednisolone (62/99, 63%) than vigabatrin (5/18, 28%, *P* = 0.01) or nonstandard treatment (2/34, 5.9%, *P* < 0.01). Of 112 infants who commenced the UKISS treatment sequence, 71/112 (63%) responded to prednisolone 40 mg/day. Among non‐responders, 12/29 (41%) subsequently responded to prednisolone 60 mg/day, and 10/22 (45%) to vigabatrin. Severe side effects and spasm relapse were not significantly different between each treatment.

**Conclusion:**

We confirm higher rates of spasm cessation with initial treatment with prednisolone than vigabatrin and nonstandard therapy. Non‐use of prednisolone as first treatment in over one third of infants highlights a concerning treatment gap. The UKISS treatment sequence has high overall treatment response (total 93/112; 83%), with similar benefit of subsequent prednisolone 60 mg/day and vigabatrin in prednisolone 40 mg/day non‐responders.

## What is already known on this topic

1. Hormone therapy (prednisolone or ACTH) and vigabatrin are effective treatments for infantile spasms (IS).
2. There is evidence from randomised control trials that hormone therapy achieves a higher rate of spasm cessation than vigabatrin as first‐line treatment for IS due to causes other than tuberous sclerosis complex.
3. In infants who do not respond to hormone therapy, treatment with vigabatrin is associated with a higher rate of spasm cessation than further hormone therapy or other antiseizure medications.

## What this paper adds

1. We showed an important treatment gap, as one third of infants did not receive first‐line treatment with prednisolone.
2. Half of the infants who did not respond to prednisolone 40 mg/day responded to the second or third steps in the UKISS treatment sequence, highlighting clinically significant incremental benefits of these steps.

Infantile spasms (IS) syndrome is the most common severe epilepsy of infancy, with numerous aetiologies, and poor developmental and seizure outcomes.^1^, ^2^, ^3^ Prompt therapy that stops IS improves long‐term outcomes.^4^, ^5^

Hormone therapy (prednisolone or adrenocorticotrophic hormone (ACTH)) and vigabatrin (VGB) are the most effective treatments for IS, and are referred to as ‘standard treatments’.^2^, ^3^, ^5^, ^6^, ^7^ Two studies have reported considerably lower rates of IS control with other antiseizure medications (nonstandard treatments).^7^, ^8^

Vigabatrin is recommended first‐line therapy for IS secondary to tuberous sclerosis complex (TSC).^9^ The United Kingdom Infantile Spasms Study (UKISS) showed that in infants without TSC, initial hormone therapy was associated with superior IS control compared to VGB, a finding later confirmed in other studies.^2^, ^4^, ^6^, ^8^, ^10^

Infants in the UKISS study randomised to the hormonal treatment arm received prednisolone or ACTH, prednisolone being preferred in Australia due to cost, availability, tolerability and ease of administration.^11^ Treatment commenced with prednisolone 40 mg/day (PNL40), escalating to 60 mg/day (PNL60) if spasms persisted after 1 week. If spasms continued after another week, vigabatrin 100–150 mg/kg/day was added while prednisolone was weaned.^2^, ^6^ This sequence (UKISS treatment sequence) has been widely implemented in Australia, although the proportion of infants receiving this sequence as compared to non‐evidence‐based treatment is not known.

Recently, the International Collaborative Infantile Spasm Study (ICISS) demonstrated that first‐line combination hormone and vigabatrin therapy has a higher rate of spasm cessation compared to hormone monotherapy.^5^ Combination therapy is not currently widely used as longer‐term outcomes have not yet been reported and concerningly, several case series have suggested that combination VGB and hormonal therapy may be associated with a higher risk of significant VGB toxicity.^12^, ^13^

Although the UKISS treatment sequence is now widely used, two things remain unknown. First, is there incremental benefit of dose escalation from PNL40 to PNL60? This was not reported in the UKISS study and evidence for the use of PNL60 is limited to a small case series in which hormone therapy was commenced at this dose.^14^ Second, the overall response to all steps of the treatment sequence has not been reported, and the only study to report response to vigabatrin after hormone treatment failure did not report doses.^7^

In this real‐world cohort study of infants with IS, we determined response to initial treatment with prednisolone, vigabatrin and nonstandard treatment to add to literature on comparative effectiveness of IS treatments and determine the proportion of infants receiving evidence‐based treatment. Additionally, we investigated the response to the UKISS treatment sequence to determine the overall response and incremental benefit of each step in the sequence.

## Methods

### Participants

We conducted a retrospective, observational study in infants with IS onset before age 18 months, without TSC, born in Victoria, Australia. Infants who did not receive treatment, or who received combination of prednisolone and vigabatrin therapy or PNL60 as first treatment, were excluded.

The study was approved by the Human Research Ethics Committees at The Royal Children's Hospital (RCH), Monash Children's Hospital, Austin Health and Geelong University Hospital.

Infants with IS were identified through questioning paediatric neurologists and reviewing electroencephalogram (EEG) reports, as previously reported.^1^ From the medical records, we obtained data on sex, aetiology, age at IS onset, EEG data, treatments received, response to treatment, side effects, and follow‐up data to age 2 years ± 3 months or last review if this was before age 2 years. Data collection occurred from January to May 2019.

### Analysis

The primary outcome was clinical response to treatment, defined as spasm cessation (no witnessed spasms for at least seven consecutive days) at 42 days following treatment and before starting another medication. Infants with missing treatment response data were excluded from analysis. Infants with documented spasm cessation after treatment who were not followed for >42 days were considered to have responded to treatment because they did not represent to the treating neurologist.

Secondary outcomes were the presence of severe side effects (defined as side effects requiring intervention, such as hospital admission, additional treatment or therapy adjustment), and spasm relapse after 42 days.

Two analyses were performed. First, we evaluated response to first IS treatment, comparing PNL40 with vigabatrin and nonstandard treatment. In the second, we considered outcomes in infants who had commenced the UKISS treatment sequence at any point (with or without prior nonstandard treatment, but with no use of vigabatrin or PNL60 prior to PNL40 – that is, PNL40 was the first standard treatment used) in order to determine incremental response to each step of the UKISS treatment sequence.

### Statistical analysis

R version 1.4 was used for statistical analysis. Differences in proportions were analysed with Pearson's Chi‐square test or Fisher's exact test where appropriate. A *P* value less than 0.05 was taken to be significant. Non‐overlapping 95% confidence intervals were considered significant for non‐mutually exclusive populations. Missing data were excluded from analysis.

## Results

### Cohort characteristics

Medical records of 170 infants with IS were reviewed. Ten infants with TSC were excluded. Three infants who received a combination of prednisolone and vigabatrin as first therapy, one infant who received PNL60 as first therapy and one infant who received no therapy were excluded. Baseline characteristics of the infants receiving prednisolone, vigabatrin and nonstandard treatment as first IS treatment were similar (Table 1). Median follow‐up from the first treatment was 14.8 months (interquartile range 9.2–18.0 months).

**Table 1:** Baseline characteristics in 155 individuals with infantile spasms

		First treatment			
	Total (*n* = 155)	PNL40 (*n* = 101)	Vigabatrin (*n* = 19)	Nonstandard (*n* = 35)	*P* value
Sex, *n* (%)					
Male	87 (56)	57 (56)	8 (42)	22 (63)	0.34
Female	68 (44)	44 (44)	11 (58)	13 (37)	
Seizures before IS, *n* (%)					
No	104 (67)	73 (72)	10 (53)	21 (60)	0.10
Yes	46 (30)	24 (24)	8 (42)	14 (40)	
Unknown	5 (3.2)	4 (4.0)	1 (5.3)	0 (0)	
Age of IS onset (months), median (IQR)	6.1 (3.8)	6.3 (3.0)	6.3 (4.0)	6.3 (5.2)	0.67
Aetiology, *n* (%)					
Structural (acquired)	25 (16)	17 (17)	2 (11)	6 (17)	0.92
Structural (malformative)	49 (32)	29 (29)	8 (42)	12 (34)	
Genetic	29 (19)	19 (19)	4 (21)	6 (17)	
Metabolic	2 (1.3)	1 (1.0)	0 (0)	1 (2.9)	
Unknown	50 (32)	35 (35)	5 (26)	10 (29)	
Hypsarrhythmia/modified hypsarrhythmia, *n* (%)					
Yes	86 (56)	61 (60)	11 (58)	14 (40)	0.15
No	68 (44)	40 (40)	8 (42)	20 (57)	
Unknown	1 (0.6)	0 (0)	0 (0)	1 (2.9)	
Time to first IS treatment (weeks), median (IQR)	2.0 (5.4)	2.8 (6.8)	0.64 (1.9)	1.7 (7.8)	0.47

IQR, interquartile range; IS, infantile spasms; PNL40, prednisolone 40 mg/day; SD, standard deviation.

### Response to first treatment

**Fig. 1:**
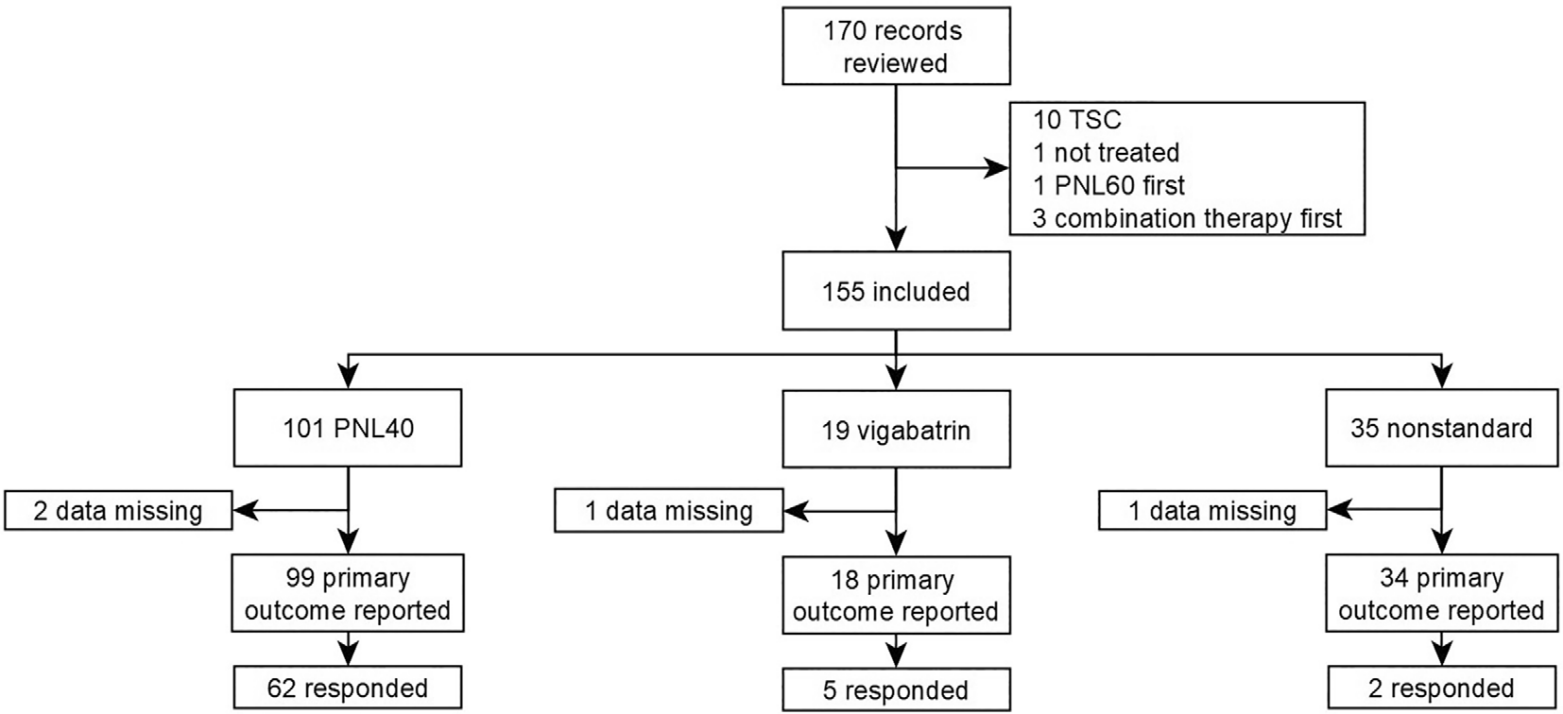
Response to initial treatment of infantile spasms. PNL40, prednisolone 40 mg/day; TSC, tuberous sclerosis complex.

101/155 (65%) infants received PNL40 as first IS treatment, 19/155 (12%) received vigabatrin and 35/155 (23%) received nonstandard treatment (Fig. 1). Data on treatment response were missing for two individuals treated with PNL40, one with vigabatrin and one with nonstandard treatment. Response to PNL40 was greater than response to vigabatrin (PNL40 62/99 (63%), vigabatrin 5/18 (28%), difference 35%, 95% CI 8.8–61%, χ ^2^ = 6.2, *P* = 0.01) and to nonstandard treatment (2/34 (5.9%), difference 57%, 95% CI 42–71%, χ ^2^ = 30, *P* < 0.01).

Of infants who responded to first treatment, one was not followed for more than 42 days. Relapse after 42 days was not significantly different between infants who responded to prednisolone compared with vigabatrin and nonstandard treatment (prednisolone 11/62 (18%, 95% CI 9.6–30%); vigabatrin 1/5 (20%, 95% CI 1.1–70%); nonstandard treatment 0/2 (0%, 95% CI 0–80%)).

### Response to UKISS treatment sequence in 115 infants

PNL40 was the first standard treatment used in 115 infants (first treatment in 101, used following a nonstandard treatment in 14); these infants were deemed to have commenced the UKISS treatment sequence. Treatment response data were missing for three individuals. Response to PNL40 was 71/112 (63%, 95% CI 54–72%) (Table 2).

**Table 2:** Primary and secondary outcomes of the United Kingdom Infantile Spasms treatment sequence in 115 individuals whose first standard therapy was prednisolone 40 mg/day

Outcome, *n*, (95% CI)	PNL40 (*n* = 115†)	PNL60 (*n* = 29)	Vigabatrin (*n* = 23‡)	PNL40 and PNL60 (*n* = 115)	Prednisolone and vigabatrin (*n* = 115)
Response	71/112 (63, 54–72)	12/29 (41, 24–61)	10/22 (45, 25–67)	83/112 (74, 65–82)	93/112 (83, 75–89)
Severe side effects	8/115 (7.0, 3.3–14)	3/29 (10, 2.7–28)	2/23 (8.7, 1.5–30)	11/115 (9.6, 5.1–17)	13/115 (11, 6.4–19)
Relapse after day 42	12/71 (17, 9.4–28)	3/12 (25, 6.7–57)	3/10 (30, 8.1–65)	15/83 (18, 11–28)	18/93 (19, 12–29)

95% CI, 95% confidence interval; PNL40, prednisolone 40 mg/day; PNL60, prednisolone 60 mg/day.

† Response data were missing for three infants in the prednisolone 40 mg/day group.

‡ Response data were missing for one infant in the vigabatrin group.

**Fig. 2:**
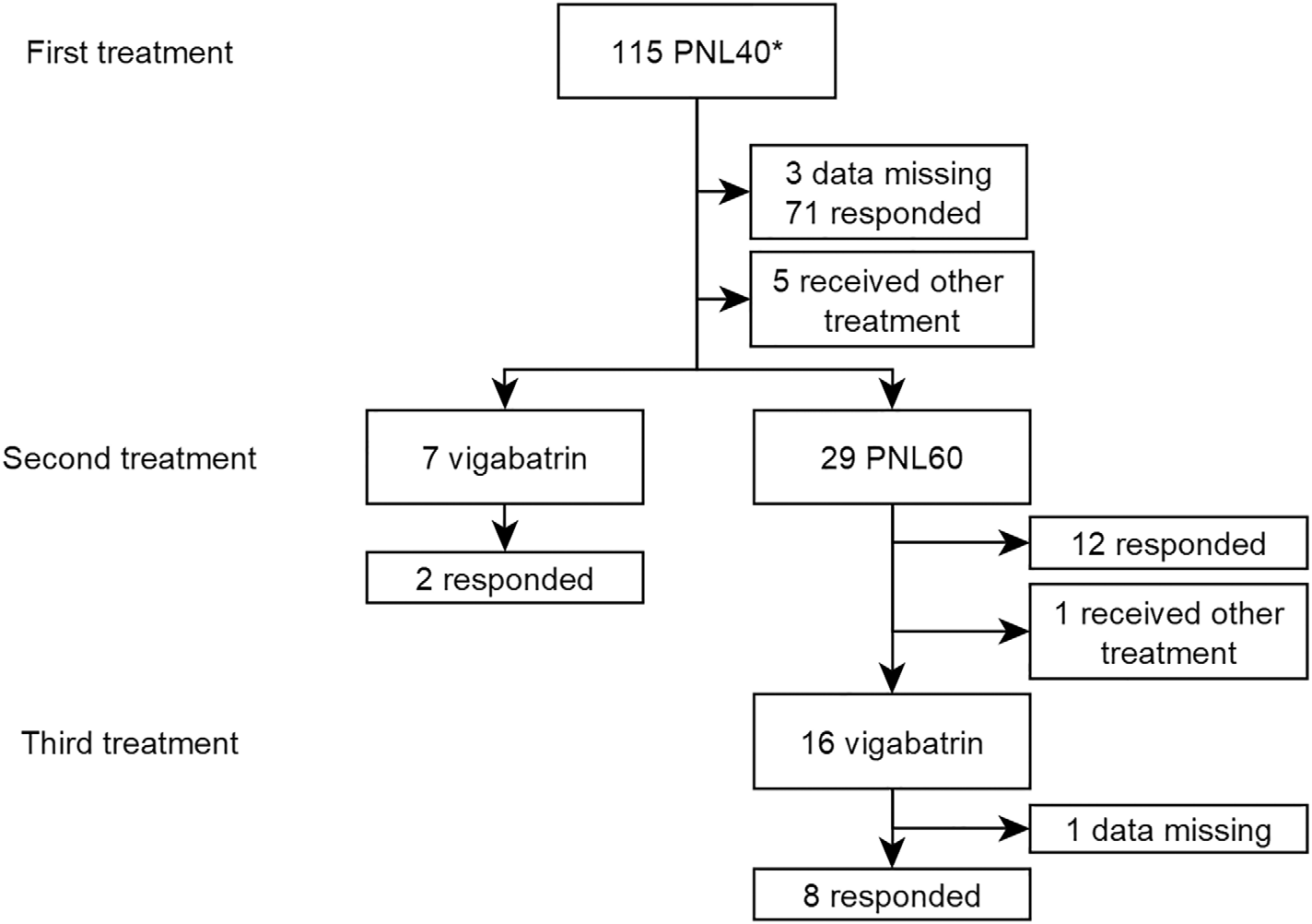
Response to the United Kingdom Infantile Spasms treatment sequence of prednisolone 40 mg, 60 mg and/or vigabatrin. PNL40, prednisolone 40 mg/day; PNL60, prednisolone 60 mg/day. †Among infants who commenced the UKISS treatment sequence at some point, the initial treatment for infantile spasms was PNL40 in 101/115 (88%) and nonstandard treatment in 14/115 (12%).

Twenty‐nine infants who did not respond to PNL40 were escalated to PNL60 (Fig. 2), with response in 12/29 (41%, 95% CI 24–61%). Therefore, dose escalation increased the overall response to prednisolone to 83/112 (74%, 95% CI 65–82).

Sixteen infants received vigabatrin following PNL40 and PNL60 treatment failure and seven received vigabatrin following PNL40 treatment failure. Treatment response data were missing for one individual. 10/22 (45%, 95% CI 25–67%) infants responded to VGB. Therefore, vigabatrin treatment following prednisolone treatment failure increased response to 93/112 (83%, 95% CI 75–89%).

Overall, 22/41 (54%) PNL40 non‐responders responded to either PNL60 or VGB, although 5/41 (12%) received neither treatment.

Three infants who responded to PNL40 were not followed up for >42 days. All infants treated with PNL60 and VGB were followed for at least 42 days. The proportion of infants who relapsed >42 days following treatment was similar for PNL40 (12/71 (17%, 95% CI 9–28%)), PNL60 (3/12 (25%, 95% CI 5.5–57%)) and vigabatrin (3/10 (30%, 95% CI 6.7–65%)). Overall, 18/93 (19%) of responders to the UKISS treatment sequence relapsed while 75/112 (67%) demonstrated a sustained treatment response.

The proportion of infants with severe side effects was similar for PNL40, PNL60 and vigabatrin (PNL40 8/115 (7%, 95% CI 3.3–14%); PNL60 3/29 (10%, 95% CI 2.7–28%); vigabatrin 2/23 (8.7%, 95% CI 1.5–30%)). One infant developed bradycardia on PNL40 resulting in cessation of therapy, five developed infections (respiratory infections and oral thrush) requiring oral antimicrobials, one developed urosepsis prompting hospital admission and one developed hypertension requiring antihypertensive medication. Three infants developed infections (cellulitis and otitis media) on PNL60 requiring antibiotics and one developed hypertension requiring treatment. One infant developed hypotonia on VGB, prompting cessation, and another became sedated, prompting dose reduction. No deaths occurred during treatment.

### Treatment response by aetiology and presence of hypsarrhythmia

There was no significant difference between aetiologic groups with respect to first treatment received (Table 1). Among infants who commenced the UKISS treatment sequence, there was no significant difference between aetiologic groups in response to PNL40 (acquired structural 12/20 (60%); malformative structural 19/33 (58%); genetic 12/20 (60%); metabolic 1/1 (100%); unknown 27/38 (71%); *P* = 0.76), PNL60 (acquired structural 4/6 (67%); malformative structural 5/12 (42%); genetic 2/4 (50%); unknown 1/7 (14%); *P* = 0.32), VGB (acquired structural 0/4 (0%); malformative structural 5/7 (86%); genetic 1/3 (33%); unknown 4/8 (50%); *P* = 0.15) or response to the UKISS sequence overall (acquired structural 16/20 (80%); malformative structural 29/33 (88%); genetic 15/20 (75%); metabolic 1/1 (100%); unknown 32/38 (84%); *P* = 0.68). Infants with a malformative structural cause had a significantly higher relapse rate (acquired structural 2/16 (11%); malformative structural 11/29 (38%); genetic 3/15 (20%); metabolic 0/1 (0%); unknown 2/32 (6%); *P* = 0.02).

EEG data were available for all infants except one whose first treatment was a nonstandard therapy. Eighty‐six infants (56%) had hypsarrhythmia/modified hypsarrhythmia (collectively referred to as hypsarrhythmia), and 68 (44%) had other EEG patterns (e.g. focal EEG abnormality). The proportion of infants with hypsarrhythmia was not significantly different between each first IS treatment (Table 1). Infants with hypsarrhythmia were significantly more likely to respond to PNL40 as first treatment compared with those without hypsarrhythmia (43/60 (72%) vs. 19/39 (49%), χ ^2^ = 4.38, *P* = 0.04). Response to VGB (3/11 (27%) vs. 2/7 (29%), *P* = 1.00) and nonstandard therapy (0/14 (0%) vs. 2/19 (11%), *P* = 0.50) was not associated with the presence of hypsarrhythmia. Of those treated with the UKISS sequence, infants with hypsarrhythmia were significantly more likely to respond to PNL40 than those without (49/67 (73%) vs. 22/45 (49%), χ ^2^ = 5.81, *P* = 0.02). The presence of hypsarrhythmia was not associated with response to PNL60 (6/13 (46%) vs. 6/16 (38%), χ ^2^ = 0.008, *P* = 0.93), VGB (4/10 (40%) vs. 6/12 (50%), *P* = 0.69), the UKISS sequence overall (59/67 (88%) vs. 34/45 (76%), χ ^2^ = 2.17, *P* = 0.14) or relapse after 42 days (12/59 (20%) vs. 6/34 (18%); χ ^2^ < 0.01, *P* = 0.97).

## Discussion

In this retrospective study of real‐world treatment of IS we report treatment response in 83% of infants who commenced the widely used UKISS treatment sequence, with sustained response in 67%.

Like previous studies, we show that first‐line treatment with prednisolone has a significantly higher response rate than vigabatrin or nonstandard treatments.^6^, ^8^ We highlight that, despite existing evidence of superiority of hormonal therapy, one third of infants in our cohort did not receive prednisolone as first‐line treatment. Further, two thirds of those infants received nonstandard treatment, which had a response rate of only 6% in our study, comparable to the 8% rate reported by Grinspan *et al*.^8^

Our findings suggest adherence to evidence‐based protocols recommending treatment with prednisolone first has not improved over the past decade.^15^ The reasons for non‐use of prednisolone as the initial treatment were not investigated in detail here. A recent study reported that pre‐existing epilepsy, and presence of a focal EEG abnormality rather than hypsarrhythmia/modified hypsarrhythmia are important factors in the choice of standard therapies although, in our study, choice of first treatment did not significantly differ with the presence or absence of hypsarrhythmia.^16^ In our study, infants with hypsarrhythmia/modified hypsarrhythmia had a higher response rate to PNL40 than those without, although the response rates to VGB and non‐standard treatments, as well as the overall UKISS treatment sequence, were comparable. Notably, the response rate to PNL40 (47%) in infants without hypsarrhythmia was still higher than to VGB (29%) and nonstandard treatment (11%), highlighting that prednisolone should not be avoided in these infants. Given prompt, effective treatment improves developmental outcomes, improving the use of evidence‐based treatment approaches in IS a priority.^4^, ^5^

Importantly, we saw clinically significant benefits of both escalation from PNL40 to PNL60 and utilisation of vigabatrin following prednisolone treatment failure, with these two steps increasing treatment response from the 63% achieved with the PNL40 step alone, without a significantly increased rate of serious side effects. Overall, half of the infants who did not respond to PNL40 responded to PNL60 or VGB. This may be an underestimate of response to these steps given five infants did not receive either treatment, and seven moved from PNL40 to VGB, skipping the PNL60 step. The combined response to PNL40 and PNL60 (74%) was comparable to reported prednisolone response in the UKISS study.^6^ While it was not possible to determine if the incremental benefit of PNL60 was due to the higher dose of prednisolone or the extended duration of treatment with PNL, it is notable that PNL60 was tolerated by most infants, providing some reassurance of its safety compared to PNL40. A similar proportion responded to PNL60 and VGB, although our study is limited in determining whether either step is inferior, due both to low numbers and to the sequential rather than head‐to‐head use of these treatments.

In studying the treatment of IS in an Australian setting, the retrospective nature of this study allowed us to audit real‐world practice and highlight an area for improvement. However, in investigating the incremental response to each step of the UKISS treatment sequence, the retrospective design is a limitation in that it reduces the ability to account for confounding factors that may have influenced treatment response. Nevertheless, baseline characteristics were comparable between infants receiving different first treatments. Despite this limitation, our cohort was similar to that of the UKISS study for non‐treatment‐related prognostic factors and overall response to PNL, suggesting our findings on response to the UKISS treatment sequence are likely applicable to other populations of infants with IS with a similar aetiologic spectrum.^5^, ^6^

It is important to note that future IS treatment recommendations may change, and favour combination therapy with hormone therapy and vigabatrin, if the ICISS trial shows that it confers long‐term developmental benefits over a sequential pathway commencing with hormone monotherapy.^6^ Identification of patient factors that predict the likelihood of treatment response may also alter future recommendations. In this study, IS aetiology was not found to be associated with prednisolone response or overall response to the UKISS treatment sequence, though sustained response to the UKISS treatment sequence beyond 42 days was lower for malformative structural aetiologies.

## Conclusion

Our findings that only two thirds of infants received evidence‐based first‐line treatment, and the clinically significant benefits of each step in the UKISS treatment sequence, highlight that improved adherence to evidence‐based protocols is a priority for improving outcomes in infants with IS.
